# Effect of Added Dietary Betaine and Soluble Fiber on Metabolites and Fecal Microbiome in Dogs with Early Renal Disease

**DOI:** 10.3390/metabo10090370

**Published:** 2020-09-15

**Authors:** Eden Ephraim, Dennis E. Jewell

**Affiliations:** 1 Pet Nutrition Center, Hill’s Pet Nutrition, Inc., Topeka, KS 66617, USA; 2 Department of Grain Science and Industry, Kansas State University, Manhattan, KS 66506, USA; djewell@ksu.edu

**Keywords:** canine, chronic kidney disease, betaine, fiber, plasma, feces, metabolomics, microbiome, body composition

## Abstract

Renal diets are recommended for dogs with chronic kidney disease (CKD). This study examined the effects of foods with added betaine and fiber on the plasma and fecal metabolome and fecal microbiome in dogs with early stage CKD. At baseline, several metabolites differed between healthy dogs and those with CKD. Dogs with CKD (n = 28) received a control food, low soluble fiber plus betaine food (0.5% betaine, 0.39% oat beta-glucan, and 0.27% short-chain fructooligosaccharides (scFOS)), or high soluble fiber plus betaine food (0.5% betaine, 0.59% oat beta-glucan, and 0.41% scFOS) each for 10 weeks in different sequences. Consumption of test foods led to several favorable, significant changes in the plasma metabolome, including decreases of several uremic toxins and other deleterious metabolites, and increases in favorable metabolites compared with the control food. Only 7 fecal metabolites significantly changed with consumption of the test foods compared with the control food, largely increases in polyphenols and lignans. Few changes were seen in the fecal microbiome, though some taxa that significantly changed in response to the test foods have beneficial effects on health, with some negatively correlating with uremic toxins. Overall, foods with added betaine and soluble fiber showed positive effects on the plasma and fecal metabolomes.

## 1. Introduction

As in humans, chronic kidney disease (CKD) is a health concern in dogs and leads to an increased risk of cardiovascular disorders, chronic inflammation, and mortality. The International Renal Interest Society (IRIS) defines stage 1 CKD in dogs as blood creatinine <1.4 mg/dL or symmetric dimethylarginine (SDMA) <18 but >14 μg/dL, along with another renal abnormality [1]. SDMA is an early indicator of CKD, and its measurement is easy to analyze and correlates with the gold standard of kidney function, glomerular filtration rate (GFR) [2,3]. Of dogs aged 7–11 years, 8–12% have elevated SDMA and/or creatinine, and the percentage increases further with age [4].

In early stage CKD in dogs, treatment focuses on slowing the progression of the disease [5]. Cats with CKD have altered metabolite and microbiome profiles compared with healthy controls [6], and several metabolites have been characterized that lead to CKD progression in humans [7]. In CKD, dysbiosis of the intestinal microbiome and its uremic metabolites damage the intestinal epithelial tight junction and its barrier function [8], leading to the systemic inflammation and higher rates of cardiovascular disease that are hallmarks of CKD [9,10].

Nutritional interventions have been shown to have beneficial effects on health-related markers, including in dogs and cats with early CKD and/or inflammation [11,12,13]. Several studies have shown anti-inflammatory properties of betaine (trimethylglycine) in addition to its roles as a methyl group donor and osmoprotectant [14,15]. In addition, supplementation with betaine reduced levels of serum uric acid and improved kidney function in a hyperuricemic mouse model [16].

The health benefits of short-chain fructooligosaccharides (scFOS), quickly fermented small inulin oligomers, have been demonstrated in multiple studies [17]. Consumption of fermentable oligosaccharides by rats led to a decrease in blood urea [18]. In humans with non-dialysis-dependent CKD, a three-month, randomized, double-blind, placebo-controlled trial showed a reduction in the microbial-derived uremic toxin p-cresyl sulfate with the consumption of fructooligosaccharide [19]. In addition, a small study in which nine overweight beagles were given inulin-type fructans found changes in metabolites and the gut microbiome [20].

Beta-glucan is a soluble fiber found in oats and barley that is well known for lowering cholesterol [21]. Oat beta-glucan has been tested as a dietary supplement in dogs, and was shown to lower total cholesterol and low and very low density lipoproteins [22]. Serum levels of the microbial uremic toxin trimethylamine N-oxide (TMAO) were significantly decreased from baseline in patients with CKD who consumed a supplement of oat beta-glucan for 12 weeks [23].

A renal diet for dogs with CKD is considered to be the standard of care, while significant benefit has been shown with earlier intervention. There is evidence that these diets can reduce biomarkers of renal insufficiency, prevent or delay the onset of uremia, and increase quality of life and long-term survival [5,12,24]. Due to the beneficial effects described above, this study evaluated the effect of added betaine as well as soluble fiber from scFOS and oat beta-glucan on body composition and levels of uremic toxins in dogs with CKD. To this end, the circulating and fecal metabolomes were assessed as well as the fecal microbiome. In addition, the baseline differences in plasma and fecal metabolites between dogs with and without CKD were compared.

## 2. Results

### 2.1. Study Design, Animals, and Food

In this study, 56 beagles were fed a control food for four weeks, after which the 28 renal insufficient dogs were randomly assigned to varying sequences of control food, test food with low soluble fiber plus betaine (0.5% betaine, 0.39% oat beta-glucan, and 0.27% scFOS), or test food with high soluble fiber plus betaine (0.5% betaine, 0.59% oat beta-glucan, and 0.41% scFOS), each for 10 weeks.

Results of the proximate analysis of the foods are presented in Table 1. As expected with the addition of oat beta-glucan and scFOS, the amount of soluble fiber increased from the control to the low soluble fiber plus betaine to the high soluble fiber plus betaine food. Since betaine does not contain fiber, and scFOS contributes very little amount to the total crude fiber, the control food does not greatly differ in crude fiber concentration compared with the test foods.

Of the dogs in the study, 28 were healthy and used for the comparison after the prefeed period (10 female, 18 male). The other 28 (18 female, 10 male) had SDMA >14 μg/dL (mean ± standard deviation, 16.4 ± 3.0 μg/dL), consistent with IRIS stage 1 CKD, and were used to investigate the effects of the renal protective foods during the study. The mean ± standard deviation age was 7.0 ± 2.7 years in the healthy group and 10.0 ± 3.8 years in the CKD group. One dog with CKD was removed from the study due to abdominal issues after completing the initial washout period and part of treatment feeding period 1 (on the control food). Otherwise, no adverse events were encountered during the study period.

There were no significant differences in body composition between the healthy and CKD groups at baseline, including in total body mass, lean body mass, total fat, and bone mineral composition (BMC). Initial body weights were 11.0 ± 3.5 kg and 10.8 ± 3.1 kg in the healthy and CKD groups, respectively. During the study, although there were numerical increases in lean body mass and decreases in total fat when dogs with CKD were fed the low soluble fiber plus betaine and the high soluble fiber plus betaine foods, none of the differences were statistically significant among food types (Table 2). Dogs with CKD had higher average daily total intake of the food containing low soluble fiber plus betaine compared to both the control (*p* < 0.001) and the high soluble fiber plus betaine (*p* = 0.002) foods.

### 2.2. Comparison of Metabolites at Baseline between Healthy Dogs and Those with CKD

From serum chemistry analyses at baseline, dogs with CKD had significantly higher creatinine (mean ± SD: 0.83 ± 0.04 mg/dL) than healthy dogs (0.66 ± 0.04 mg/dL; *p* = 0.002) as well as higher blood urea nitrogen (14.7 ± 1.1 mg/dL) than healthy dogs (8.3 ± 1.1 mg/dL; *p* = 0.0001). There were no significant differences between groups in other non-metabolite analytes, urine specific gravity, or urine pH at baseline.

**Figure 1:**
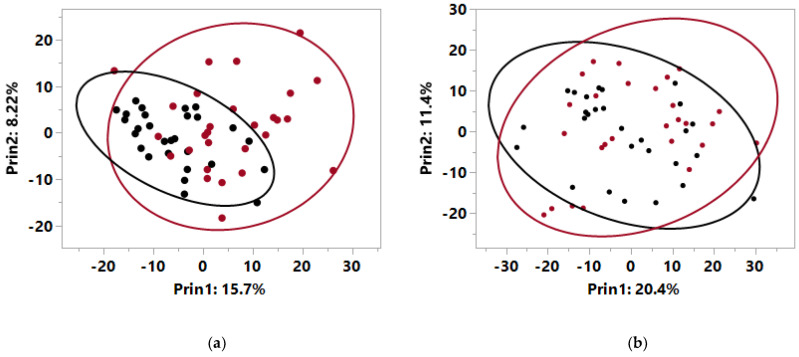
Principal components analysis for metabolomics data at baseline from (**a**) plasma and (**b**) feces from healthy dogs (black) and dogs with early stage renal disease (red) with circles indicating 95% confidence intervals.

A total of 722 metabolites were detected via metabolomics analysis in plasma at baseline from both healthy dogs and those with CKD. Principal components analysis (PCA) showed that the metabolites with high values on principal component 1 (PC1) had a significant contribution to the separation of the normal dogs from the dogs with CKD (*p* < 0.0001 for PC1 and *p* = 0.2 for PC2; Figure 1a, Table S1). Levels of 31 circulating metabolites were significantly different at baseline between the healthy dogs and those with CKD (Table 3). In particular, dogs with CKD had higher levels of creatinine, urea, indoles, advanced glycation end products (AGE), and other uremic toxins of microbial origin. Conversely, dogs with CKD had significantly lower levels of monounsaturated (MUFA) and polyunsaturated fatty acids (PUFA) such as tetradecadienoate, palmitoleate, docosatrienoate, and 5-dodecenoate compared with healthy dogs.

Fecal measurements, including pH, ash, and moisture, were not significantly different between dog groups at baseline. Fecal metabolomics data at baseline identified 894 metabolites. PCA of these data showed no separation of the 95% confidence areas for healthy and CKD dogs, and that neither PC1 or PC2 were significantly different by canine health (Figure 1b). Only 6 fecal metabolites significantly differed between healthy dogs and those with CKD at baseline (Table 3).

### 2.3. Effect of Added Betaine and Fiber on Plasma Metabolites in Dogs with CKD

Following consumption of the high soluble fiber plus betaine food by dogs with CKD, 10 plasma metabolites that differed at baseline between dogs that were healthy and those with CKD significantly changed from baseline toward a healthier profile (Table 4). An additional two metabolites that were numerically higher in dogs with CKD at baseline, 4-hydroxyphenylacetate and dihydrocaffeate sulfate (*p* = 0.089 and *p* = 0.081, respectively), also significantly shifted after consumption of the high soluble fiber plus betaine food. Three plasma metabolites significantly changed from baseline with consumption of the low soluble fiber plus betaine food.

**Figure 2:**
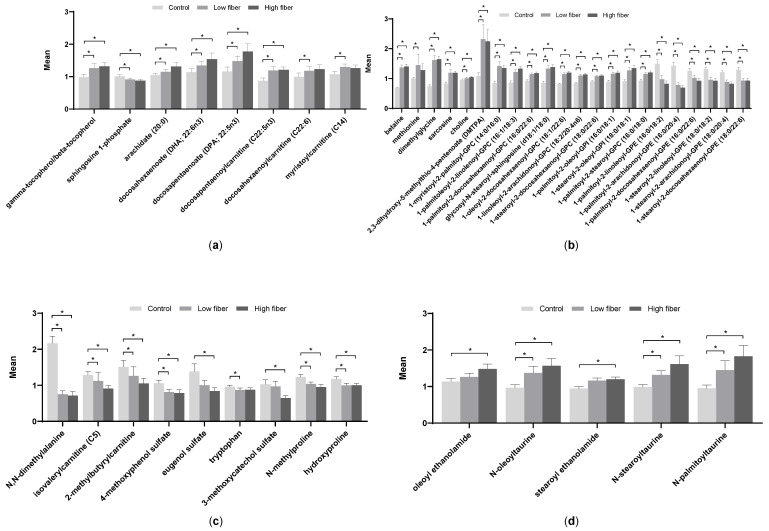
Plasma metabolites from dogs with early renal disease fed the control, low soluble fiber plus betaine, and high soluble fiber plus betaine foods. (**a**) Markers of inflammation and polyunsaturated fatty acids, (**b**) indicators of methylation, (**c**) uremic toxins and collagen breakdown products, and (**d**) endocannabinoids. Data were analyzed via a linear mixed model with animal ID as a random variable, were rescaled to a median value of 1, and are presented as group means and standard errors. * *p* ≤ 0.05. GPC, glycerophosphocholine; GPE, glycerophosphoethanolamine; GPI, glycerophosphoinositol.

In dogs with CKD, both test foods resulted in significant increases in the antioxidant markers gamma/beta-tocopherols compared with the control food (Figure 2a). In addition, both test foods significantly reduced the inflammatory marker sphingosine-1-phosphate (S1P) compared to the control food. Long-chain PUFAs, including docosahexaenoate (DHA) and docosapentaenoate (DPA), were significantly increased with one or both of the test foods compared with the control food.

Not surprisingly, due to the inclusion of betaine in the test foods, its levels were higher in plasma in dogs who consumed the test foods compared with the control food (Figure 2b). Higher levels of betaine also appeared to result in an increase in one-carbon metabolism, as evidenced by the increases in dimethylglycine (DMG) and methionine. Levels of several phosphatidylcholines and phosphatidylinositols were also significantly increased with the test foods compared with the control food. In contrast, a few phosphatidylethanolamines were significantly decreased in the test foods compared with the control food.

Levels of several uremic toxins, including 4-methoxyphenol sulfate, eugenol sulfate, and 3-methoxycatechol sulfate, were decreased by one or both of the test foods compared with the control food (Figure 2c). Levels of 2-methylbutyrylcarnitine, a branched-chain amino acid that was increased in dogs with CKD at baseline, were decreased with consumption of the high soluble fiber plus betaine food compared with the control food. In addition, both test foods resulted in significantly decreased levels of the collagen degradation product hydroxyproline as well as N-methylproline. Serum chemistry, performed separately from the metabolomic analysis, showed that creatinine significantly decreased following consumption of each of the test foods compared with the control food (Table 2).

In contrast to the uremic toxins, levels of several endocannabinoids were increased with consumption of the test food(s) compared with the control food (Figure 2d).

### 2.4. Correlations of Plasma Metabolites with Known Markers of CKD

Plasma metabolites following consumption of the test foods were correlated with serum creatinine and serum SDMA in order to measure the strength of their relationships (Table 5). Many known uremic toxins were significantly positively correlated with these two markers of CKD, including creatinine, urea, and TMAO. Several of these metabolites were also present at higher levels in dogs with CKD versus healthy dogs as shown in Table 3. Conversely, a negative correlation was observed with MUFA and PUFA, which were also increased in healthy dogs relative to those with CKD in Table 3, as well as with several omega-3 fatty acids.

### 2.5. Effect of Added Betaine and Fiber on Fecal pH and Metabolites in Dogs with CKD

Fecal pH, moisture, and ash were similar among groups after the treatment period (Table 6). At the end of the feeding treatment period, dogs with CKD exhibited significant changes from baseline in several fecal metabolites that differed at baseline between healthy dogs and those with CKD (Table 7). The polyphenol genistein sulfate increased from baseline with both the test foods.

**Figure 3:**
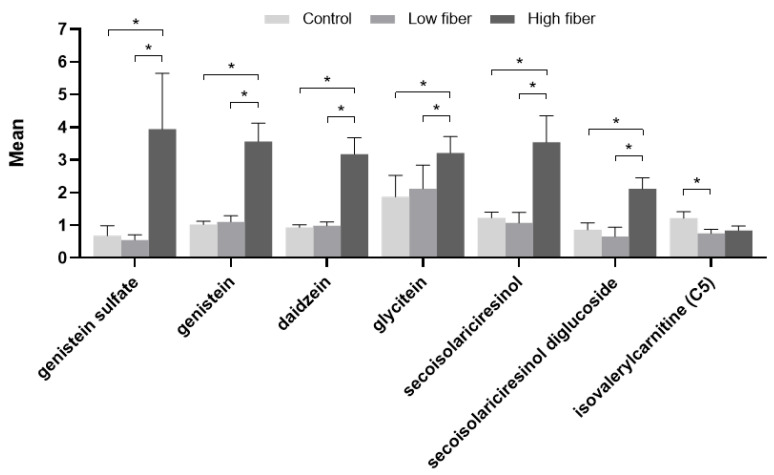
Fecal metabolites from dogs with early renal disease fed the control, low soluble fiber plus betaine, and high soluble fiber plus betaine foods. Data were analyzed via a linear mixed model with animal ID as a random variable, were rescaled to a median value of 1, and are presented as group means and standard errors. * *p* ≤ 0.05.

In comparing the effects of consumption of the control and test foods in dogs with CKD, those fed the high soluble fiber plus betaine food showed significant increases relative to the control and low soluble fiber plus betaine foods in the polyphenols genistein sulfate, genistein, daidzein, and glycitein, as well as the lignan secoisolariciresinol and its precursor, secoisolariciresinol diglucoside (SDG), in the fecal metabolome (Figure 3). A significant decrease in isovalerylcarnitine was seen with the low soluble fiber plus betaine test food compared with the control food, with a nonsignificant numerical decrease with the high soluble fiber plus betaine test food.

### 2.6. Effect of Added Betaine and Fiber on the Fecal Microbiome in Dogs with CKD

Three hundred three operational taxonomic units (OTUs) met the criteria to be considered for statistical analysis. At study baseline, there were no significant differences in alpha or beta diversity between fecal samples from the healthy dogs and those with CKD. There were also no significant differences in the relative abundance of OTUs in the fecal microbiome between healthy dogs and those with CKD.

Following the treatment food periods, no significant differences in alpha or beta diversities of the fecal microbiome were observed among the dogs with CKD who consumed the control, low soluble fiber plus betaine, or high soluble fiber plus betaine foods. Only 5 OTUs showed significant differences between food types in dogs with CKD, many of which were differences between the test foods. OTU 23706 (genus *Collinsella* of phylum Actinobacteria) showed a significant decrease in the low soluble fiber plus betaine test food compared with the control food (*p* = 0.02) and the high soluble fiber plus betaine test food (*p* = 0.013). Another OTU of the phylum Actinobacteria (OTU 249375, unclassified class) was also of significantly lower abundance with the low soluble fiber plus betaine versus the high soluble fiber plus betaine test food (*p* = 0.003). The abundance of the beneficial OTU 1030652 (phylum Bacteroidetes, genus *Odoribacter*) was significantly different among all three foods, with the highest levels with the low soluble fiber plus betaine food and the lowest with the high soluble fiber plus betaine food. The fecal samples from dogs fed the control food and low soluble fiber plus betaine test food were similar in the abundance of OTU 945478 (phylum Proteobacteria, class Gammaproteobacteria, unclassified order), but the high soluble fiber plus betaine food showed significantly lower abundance compared with the low soluble fiber plus betaine food (*p* = 0.013). Similarly, the abundance of OTU 586453 (phylum Firmicutes, family Christensenellaceae) was significantly increased with the high soluble fiber plus betaine food compared with the low soluble fiber plus betaine food (*p* = 0.001).

Pearson correlation analysis showed that two plasma metabolites, the microbial metabolite 4-vinylguaiacol sulfate and the tryptophan-derived indolelactate, both negatively correlated with *Odoribacter* (Table 8). In addition, 3-methyoxycatechol sulfate and catechol sulfate negatively correlated with Christensenellaceae (OTU 586453).

Of the fecal metabolites, the phenolic 4-hydroxybenzoate and the tyrosine derivative indolepropionate negatively correlated with the unclassified Actinobacteria (OTU 249375; Table 8). Indolin-2-one negatively correlated with Christensenellaceae (OTU 586453). Conversely, two OTUs of the phylum Actinobacteria (OTU 249375, unclassified and OTU 23706, *Collinsella*) positively correlated with the lignan secoisolariciresinol, with OTU 249375 also correlating with its precursor, SDG.

## 3. Discussion

Reduced clearance of certain toxic metabolites by the kidneys in CKD leads to their accumulation and designation as uremic toxins. In this study, many known uremic toxins and other metabolites that are increased in patients with CKD, such as ADMA/SDMA, creatinine, urea, hippurate, AGE, and TMAO [25,26,27,28], were detected as significantly increased in dogs with CKD compared with healthy dogs at baseline. As expected, higher creatinine and BUN levels were also observed in dogs with CKD versus healthy dogs at baseline.

**Figure 4:**
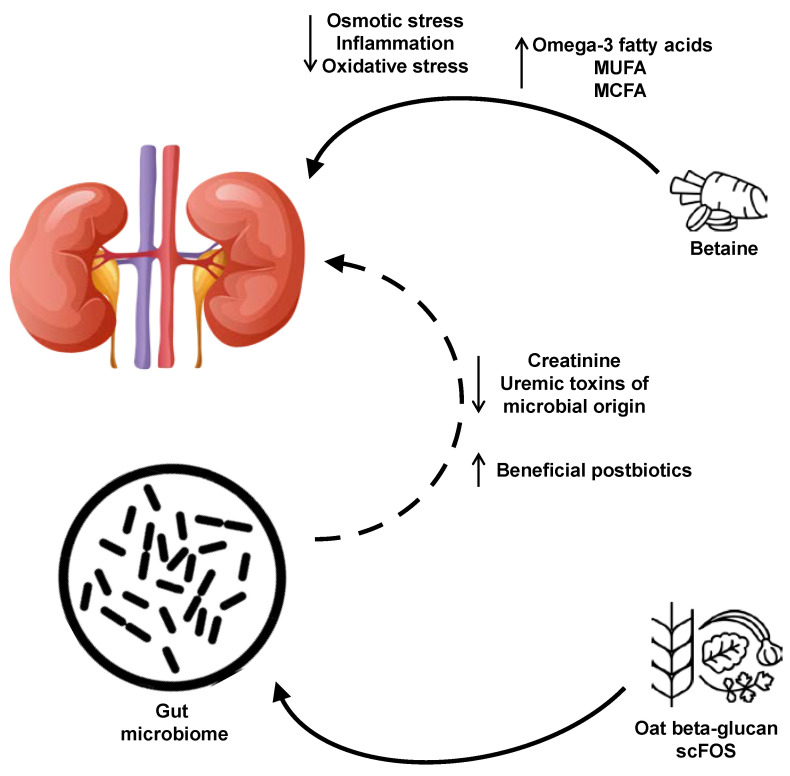
Summary of the effects of the food with added betaine, scFOS, and oat beta-glucan on metabolites and health. Consumption of oat beta-glucan and scFOS results in positive, gut microbiota-mediated changes in the metabolome, while betaine has a more direct effect on kidney health. MCFA, medium-chain fatty acids; MUFA, monounsaturated fatty acids; scFOS, short-chain fructooligosaccharides.

Overall, dogs with CKD fed either a low soluble fiber plus betaine or high soluble fiber plus betaine test food with betaine displayed a shift in plasma and fecal metabolites that indicated a healthier profile compared with those consuming the control food as summarized in Figure 4. In plasma, levels of the antioxidants gamma-tocopherol/beta-tocopherol [29] and omega-3 fatty acid DHA, known to confer cardiovascular benefits [30], were increased with the test foods. Carnitine conjugates of long-chain fatty acids, which are then transported to the mitochondria for β-oxidation [31], were also increased with the test food(s) compared with the control food. In contrast, the sphingolipid metabolite sphingosine-1-phosphate, which is involved in a number of signaling pathways including inflammation [32], was decreased with the test foods. Levels of serum creatinine, a known marker of renal disease, also significantly decreased following consumption of the test foods relative to the control food, though all values were in the normal range.

Several plasma metabolites correlated with serum creatinine and serum SDMA, many of which, including N6,N6,N6-trimethyllysine, pseudouridine, N-acetylphenylalanine, isobutyrylcarnitine, 2-methylbutyrylcarnitine, 4-hydroxyhippurate, 5-hydroxyhippurate, 3-indoxyl sulfate, and citrulline, were previously observed to be increased in patients on hemodialysis [26,27], further supporting the role of these metabolites as markers of renal disease. When the metabolite group was taken as a whole, there was a closer (higher average r) relationship overall with SDMA than creatinine. This suggests that the metabolites were, like SDMA, responding to renal decline at an earlier time than circulating creatinine.

Since betaine was added to the test foods in this study, it was expected that plasma betaine levels would be increased with the test foods compared with the control food. The observed increases in DMG and methionine with the test foods may be explained by betaine-homocysteine S-methyltransferase-mediated demethylation of betaine and dehydrogenation of homocysteine to yield DMG and methionine. Subsequently, DMG is converted to sarcosine, the levels of which were also increased with the test foods in this study. The increases in glycerophospholipids with the test foods may be due to the addition of betaine and its involvement in methylation [33].

The test food(s) led to decreases in several likely deleterious metabolites. Levels of isovalerylcarnitine, one of the metabolites that was increased in the plasma of dogs with CKD relative to healthy dogs at baseline, decreased in both the plasma and feces of dogs with CKD with consumption of the test food(s); it also significantly decreased from baseline in the plasma of dogs that consumed either test food. Isovalerylcarnitine was identified as a metabolite associated with protein intake in patients with CKD [34]. Like isovalerylcarnitine, levels of the branched-chain amino acid 2-methylbutyrylcarnitine was higher in the plasma of dogs with CKD than healthy dogs at baseline and decreased with the test foods, both compared with baseline and with consumption of the control food. In addition, it was one of four metabolites associated with a higher estrogen receptor-positive breast cancer risk [35]. The uremic solutes 4-methoxyphenol sulfate, eugenol sulfate, and 3-methoxycatechol sulfate decreased with the high soluble fiber plus betaine food compared with the control food in this study, and were previously observed to be increased in hemodialysis patients compared with controls [26].

Several other plasma metabolites were favorably affected by the test food(s). Significant increases from baseline in MUFA and PUFA were observed following consumption of the test foods; these also negatively correlated with serum creatinine and SDMA. In addition, the significant decreases in hydroxyproline, a major component of collagen, with the test foods compared with the control food may indicate decreased degradation of collagen, the most abundant protein in connective tissues. N-methylproline, a fibrosis biomarker [36], was also significantly decreased after consumption of the test foods. The levels of several endocannabinoids increased with the test foods, which are of interest since the endocannabinoid system is important in normal renal function [37].

In this study, relatively few fecal metabolites changed with consumption of the test foods. A similar study that tested food similar to the high soluble fiber plus betaine food in cats with CKD stage 1–3 also found few changes in fecal metabolites [38], several of which (glycitein, genistein sulfate, and daidzein) were changed in both studies. The sulfate conjugate of genistein, genistein sulfate, was decreased in the feces of dogs with CKD at baseline, significantly increased from baseline following consumption of the test foods, and was significantly increased in the high soluble fiber plus betaine test food compared with the control food in the present study, indicating a shift toward a healthier profile in the activity of the microbiota in dogs with CKD. The soy flavonoids increased with the test food(s) in this study, including daidzein, genistein, and glycitein, have been associated with a number of positive health attributes, including antioxidant, anticancer, and anti-inflammatory properties [39,40,41]. There is also some evidence that genistein can modulate the gut microbiome to reduce tumor growth [42]. In addition, consumption of soy protein has been shown to slow the decline of estimated GFR and decrease proteinuria in patients with CKD [43].

The lignans secoisolariciresinol and SDG also exhibited increases in the feces of dogs fed the test foods compared to the control food. SDG has been shown to confer protective effects for a range of diseases and conditions, including cardiovascular and kidney disorders, some of which are attributed to its antioxidant effects [44,45].

The gut microbiome is known to modulate the host metabolome. In this study, the few significant changes in OTUs among the control and the low soluble fiber plus betaine and high soluble fiber plus betaine test foods may reflect the relatively short treatment periods (10 weeks). Another possible explanation is that the metabolic capacity of the microbiome, not necessarily the composition of the microbiome, is more important for influencing the host metabolome [46]. Several studies have shown limited effects of consumption of increased whole plant foods, including fiber, on gut microbial composition in humans [47]. Of note, there were no significant differences between healthy dogs and those with CKD in the fecal microbiome at baseline, consistent with the few changes observed in a similar study on cats [6]. Thus, it appears that gut microbial composition in the early stages of CKD in cats or dogs is not substantially different from the healthy state.

Nevertheless, some of the taxa in the fecal microbiome that significantly changed in response to the test food(s) have beneficial effects on health. The abundance of bacteria in the genus *Collinsella* increased in the presence of inulin-type fructans, inulin, and whole fiber [48,49,50]. Here, abundance of *Collinsella* significantly decreased with the low soluble fiber plus betaine test food compared with the control and high soluble fiber plus betaine foods. However, the mechanisms behind the effect of the low soluble fiber plus betaine but not the high soluble fiber plus betaine food remain to be explored.

The role of *Odoribacter* in regard to health remains puzzling, particularly since divergent effects of the test foods were observed in this study. *Odoribacter* is a producer of butyrate, which is the preferred energy source for colonocytes, augments intestinal barrier function, and modulates immune responses [51]. Butyrate also activates the aryl hydrocarbon receptor, a regulator of colonic stem cell proliferation, barrier cell function, and immune cells, in human intestinal epithelial cells [52]. *Odoribacter* were among the genera decreased in mice with type 2 diabetes compared with normal mice, and their abundance was restored with polysaccharide-rich extracts from leaves from the shrub *Apocynum venetum* [53]. The uremic toxin 4-vinylguaiacol sulfate, which was increased in hemodialysis patients compared with healthy controls [26], was negatively associated with *Odoribacter* in the present study, also indicating a positive health effect of *Odoribacter*. However, higher abundance of *Odoribacter* has been observed in stool samples from both mice and humans with cancer [54,55]. Consistent with the observed decrease of *Odoribacter* with the high soluble fiber plus betaine test food, a prior study found that higher fiber intake was associated with significantly decreased abundance of *Odoribacter* in humans [56]. Thus, it may be that the abundance of *Odoribacter* alone does not denote a clear indication for health, but rather its interactions with other bacteria and metabolites, which also vary in different states of health and disease, are the determinant.

The family Christensenellaceae has been linked to metabolic health and correlates with a healthy gut [57]. It is enriched in people with a normal body mass index compared with obese individuals and decreased in those with metabolic syndrome and inflammatory bowel disease compared with healthy controls [57]. In senior dogs, Christensenellaceae has been reported to increase in dogs fed increased soluble fiber and omega-3 fatty acids and to negatively correlate with fecal concentrations of the AGE pyrraline and various fecal amino acids [58]. Like *Odoribacter*, it is a butyrate producer and the test foods had opposite effects on its abundance in this study. Paradoxically with the seemingly healthy effects of Christensenellaceae, it was negatively correlated with catechol sulfate, a metabolite that was negatively associated with net endogenous acid production in adults with CKD [59]. This acid production is associated with an elevated risk of CKD and faster decline in GFR.

The unclassified Actinobacteria (OTU 249375) may have a positive effect on health due to its correlations with certain plasma and fecal metabolites. This study found a negative correlation with indoles as well as with 4-hydroxybenzoate, the latter of which has been associated with cancers of the kidney [60] and stomach, with high levels associated with a worse survival rate for the latter [61]. In addition, this OTU was positively correlated with the aforementioned lignans secoisolariciresinol and SDG.

A limitation of this study is that the dogs were in the early stages of CKD and thus there were not many differences in uremic toxins compared with healthy dogs. Nevertheless, many positive changes in metabolites were observed in dogs with early CKD who consumed the betaine and fiber-enriched foods. Indeed, perhaps more positive changes in metabolites and/or decreased rates of CKD progression may be observed with a longer treatment period than the 10 weeks in this study.

In summary, this study showed an overall positive effect of the test foods composed of betaine and fiber from scFOS and beta-glucan on plasma and fecal metabolites in dogs with stage 1 CKD. Levels of several uremic toxins decreased with the test food(s) compared with the control food, and some of these also correlated with gut taxa. Future studies could evaluate the more long-term effects on health as well as progression of CKD.

## 4. Materials and Methods

### 4.1. Study Foods

The control food in this study was a complete and balanced dry food designed to aid in the management of renal disease with no added betaine or fiber from scFOS or beta-glucan. The test foods were of similar formulation to the control food except that each was supplemented with 0.5% betaine; the low soluble fiber plus betaine test food also had 0.39% oat beta glucan and 0.27% scFOS and the high soluble fiber plus betaine one had 0.59% oat beta-glucan and 0.41% scFOS. All foods contained fiber from rice, barley, and sorghum as well as 4.9% beet pulp, with the amount of rice adjusted to accommodate for the added betaine, scFOS, and oat beta-glucan. All foods were in dry form and met the Association of American Feed Control Officials (AAFCO) maintenance nutrition requirements through testing, and dietary analysis was carried out as previously described [6].

### 4.2. Animals and Experimental Design

Fifty-six beagle dogs, ranging in age from 2–15 years, all spayed or neutered and owned by Hill’s Pet Nutrition, Inc., were included. For the healthy group (n = 28), dogs with diseases, including compromised kidney and intestinal function (e.g., IBD, colitis), food allergy, and/or receipt of antibiotics or vaccines in less than a month before the start of this study were excluded. Dogs with early CKD (n = 28) had SDMA > 14 μg/dL (International Renal Interest Society stage 1 [1]), and those with disease other than kidney disease were excluded. Dogs were to be removed from the study if they would benefit from removal as determined by the colony veterinarian. All dogs were housed in pairs at the Hill’s Pet Nutrition Center, where they could interact with each other and received daily opportunities to run outside and play with toys and caretakers. Dogs were fed once daily and had unlimited access to water. At the end of the study, all dogs were returned to the colony at Hill’s Pet Nutrition.

**Figure 5:**
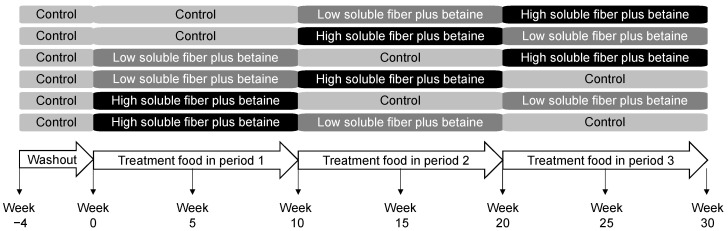
Study design and timeline in which dogs consumed the control, low soluble fiber plus betaine, and high soluble fiber plus betaine foods in a Williams Latin Square sequence.

The study protocol was approved by the Hill’s Institutional Animal Care and Use Committee (IACUC) (Permit number: 831) and Animal Welfare Committee. This study complied with the US National Research Council guide for the care and use of laboratory animals [62]. After a 4-week washout period on the control food, both groups of dogs were assigned into six subgroups to receive the control food, the low soluble fiber plus betaine test food, or the high soluble fiber plus betaine test food, each for 10 weeks, in a Williams Latin Square design sequence with age and sex balanced among groups (Figure 5). DEXA measurements were conducted using the DXA-QDR-4500 system (Hologic, Waltham, MA, USA) at the end of each treatment period. Blood and fecal samples were collected midway and at the end of each treatment period. Feces were collected within 30 min of defecation, homogenized in a Thinky Mixer (Thinky USA, Inc., Laguna Hills, CA, USA), and further processed as previously described [63]. A total of 156 fecal and 182 blood samples were successfully collected for analyses.

### 4.3. Metabolite Analysis

A chemistry screen was used to assess circulating blood creatinine and blood urea nitrogen after fasting [64]. Serum concentrations of SDMA were measured by a commercial laboratory (IDEXX Laboratories, North Grafton, MA, USA) using liquid chromatography mass spectroscopy. Analysis of plasma and fecal metabolomics was performed by Metabolon (Morrisville, NC, USA) as previously described [63].

### 4.4. Fecal Microbiome Analysis and Bioinformatics Processing

The Qiagen MagAttract Power Microbiome DNA/RNA EP DNA isolation kit (Qiagen Cat. No. ID:27500-4-EP, Germantown, MD, USA), optimized for use with the Eppendorf epMotion 5075 TMX platform (Eppendorf, AG, Hamburg, Germany), was used to extract total DNA from frozen fecal samples. PCR amplification spanned the V3-V4 hypervariable regions of the 16S rRNA gene, the Illumina library preparation protocol (15044223 Rev. A) was used for amplicon sequencing, sequences were de-multiplexed to obtain FASTQ Files, and the GreenGenes reference taxonomy at the genus level was used for bacterial taxonomic classification as previously described [63]. For microbiome data, copy numbers of the 16S genes in microbial taxa were corrected and numerical values were centered log-ratio (CLR) transformed to enable appropriate statistical analysis.

### 4.5. Statistical Analysis

The number of dogs with CKD for enrollment was estimated by performing a power analysis using alpha = 0.05, beta = 0.1, an expected difference of 0.45 between test and control foods for creatinine, and a coefficient of variation percent of 0.5, which gave 28 dogs. An equal number of control dogs (n = 28) was used to compare baseline differences between the healthy dogs and those with CKD.

JMP Pro software (JMP, Cary, NC, USA) was used for data analyses. Metabolomics data was log transformed before statistical analysis. Mean units for metabolites were scaled to a median of 1. Paired t-tests were used to compare levels of metabolites at baseline between healthy and CKD groups. Mixed modeling was used to compare the treatment effect during the treatment period using animal ID as random designate. In all analyses, statistical significance was considered to be *p* ≤ 0.05.

Body composition results from DEXA analysis were analyzed using SAS PROC MIXED analysis for repeated measures using animal ID as random designate and initial body composition as a covariate.

Fecal microbiome data were processed as previously described [6]. Regression analyses using Pearson’s correlation coefficient (r) were used for correlations between plasma metabolites and serum creatinine and SDMA as well as for OTUs and metabolites.

Alpha diversity of the fecal microbiome was evaluated as the Shannon index and inverse of the Simpson index using the R vegan package [65]. Beta diversity was compared via PERMANOVA on Bray–Curtis dissimilarity measures of relative abundances of OTUs.

## Figures and Tables

**Table 1:** Proximate analysis of the three study foods.

	Study Foods		
	Control	Low Soluble Fiber plus Betaine	High Soluble Fiber plus Betaine
**Proximate analysis**			
Moisture	9.0	8.8	9.1
Ash	3.6	3.7	3.5
Crude fat	19.2	20.4	20.0
Crude protein	15.7	15.9	14.8
Crude fiber	1.6	1.7	1.8
Neutral detergent fiber	4.4	4.4	5.1
Soluble fiber	1.9	2.0	2.1
Total fiber	7.5	7.5	7.9
Lysine	1.53	1.34	1.37
Methionine	0.64	0.63	0.58
Cystine	0.26	0.25	0.24
Phosphorus	0.34	0.34	0.33
Potassium	0.64	0.65	0.62
Chloride	0.62	0.64	0.59
Calcium	0.63	0.65	0.61
Magnesium	0.08	0.08	0.09
Betaine	0.02	0.50	0.50
Choline, ppm	2250	2236	2232
Food metabolic energy, kcal/kg	4047	4044	4043

Reported values are percentages unless otherwise indicated.

**Table 2:** Body composition parameters and average food intake in dogs with chronic kidney disease (CKD).

	Study Foods			
	Control	Low Soluble Fiber plus Betaine	High Soluble Fiber plus Betaine	*p*-Value
Total body mass, g	11,583.9 ± 608.4	11,482.9 ± 645	11,482.7 ± 587.1	0.59
Lean body mass, g	6490.1 ± 420.7	6532.0 ± 464	6525.1 ± 440.2	0.56
Total fat, g	4666.0 ± 224.3	4520.8 ± 215.3	4531.9 ± 193.4	0.71
Bone mineral composition, g	427.8 ± 22.2	430.1 ± 23.8	425.7 ± 22.1	0.59
Average intake, g/day	160.5 ± 0.8	164.9 ± 0.9	161.5 ± 0.7	* <0.001 ^†^ 0.620 ^‡^ 0.002
Creatinine, mg/dL	0.8 ± 0.1	0.7 ± 0.1	0.7 ± 0.2	* 0.004 ^†^ 0.008 ^‡^ 0.790
SDMA, μg/dL	16.0 ± 3.6	15.6 ± 3.3	15.9 ± 3.5	0.890
Total protein, g/dL	5.5 ± 0.3	5.5 ± 0.3	5.5 ± 0.3	* 0.430 ^†^ 0.046 ^‡^ 0.230
BUN, mg/dL	14.0 ± 6.7	13.3 ± 6.0	12.2 ± 5.2	0.290

* Control vs. low soluble fiber plus betaine; ^†^ Control vs. high soluble fiber plus betaine; ^‡^ low soluble fiber plus betaine vs. high soluble fiber plus betaine; BUN, blood urea nitrogen; SDMA, symmetric dimethylarginine.

**Table 3:** Differences in plasma and fecal metabolites between healthy dogs and dogs with early renal disease at baseline.

	Healthy Dogs	CKD Dogs	Difference of Log-Transformed Data	
	Mean ± SE	Mean ± SE	Healthy-Renal ± SE	*p*-Value
**Plasma metabolites**				
Acetylated peptides				
phenylacetylglycine	1.07 ± 0.11	2.12 ± 0.38	−0.55 ± 0.17	0.003
phenylacetylglutamine	0.96 ± 0.11	1.79 ± 0.34	−0.51 ± 0.19	0.010
Advanced glycation end-product				
N6-carboxymethyllysine	0.83 ± 0.05	1.38 ± 0.12	−0.47 ± 0.09	<0.001
Benzoate metabolism				
hippurate	0.94 ± 0.16	4.93 ± 1.48	−1.14 ± 0.32	0.001
4-hydroxyhippurate	0.24 ± 0.01	0.88 ± 0.25	−0.65 ± 0.20	0.004
4-acetylphenyl sulfate	0.99 ± 0.18	1.75 ± 0.27	−0.64 ± 0.23	0.008
Creatine metabolism				
creatinine	0.99 ± 0.03	1.12 ± 0.04	−0.12 ± 0.04	0.007
Fatty acid, branched-chain amino acid metabolism				
propionylglycine (C3)	0.90 ± 0.11	1.66 ± 0.18	−0.63 ± 0.14	<0.001
Food component/plant				
pyrraline	0.61 ± 0.10	1.83 ± 0.34	−0.98 ± 0.20	<0.001
indoleacetylalanine	0.48 ± 0.12	1.39 ± 0.28	−1.01 ± 0.29	0.001
ferulic acid 4-sulfate	0.63 ± 0.15	1.32 ± 0.26	−0.81 ± 0.27	0.005
2-oxindole-3-acetate	2.92 ± 1.03	5.71 ± 1.34	−1.06 ± 0.37	0.006
dihydroferulic acid sulfate	1.18 ± 0.29	3.42 ± 0.88	−0.88 ± 0.32	0.008
Leucine, isoleucine, valine metabolism				
isovalerylcarnitine (C5)	1.10 ± 0.06	1.31 ± 0.07	−0.17 ± 0.08	0.036
2-methylbutyrylcarnitine (C5)	1.08 ± 0.07	1.41 ± 0.12	−0.22 ± 0.11	0.041
Long-chain polyunsaturated fatty acid (n3 and n6)				
tetradecadienoate (14:2)	1.18 ± 0.08	0.90 ± 0.07	0.27 ± 0.11	0.019
docosatrienoate (22:3n6)	1.01 ± 0.13	0.60 ± 0.08	0.51 ± 0.20	0.013
Lysine metabolism				
N6,N6,N6-trimethyllysine	0.92 ± 0.03	1.18 ± 0.05	−0.24 ± 0.05	<0.001
N6,N6-dimethyllysine	0.89 ± 0.03	1.10 ± 0.05	−0.19 ± 0.06	0.001
Medium-chain fatty acid				
5-dodecenoate (12:1n7)	1.48 ± 0.15	0.85 ± 0.10	0.58 ± 0.18	0.002
palmitoleate (16:1n7)	1.15 ± 0.09	0.83 ± 0.07	0.32 ± 0.13	0.018
Phenylalanine metabolism				
N-acetylphenylalanine	0.80 ± 0.04	1.23 ± 0.09	−0.40 ± 0.09	<0.001
1-carboxyethylphenylalanine	0.70 ± 0.04	1.11 ± 0.12	−0.38 ± 0.12	0.003
2-hydroxyphenylacetate	0.99 ± 0.09	1.24 ± 0.08	−0.29 ± 0.12	0.019
Phospholipid metabolism				
trimethylamine N-oxide	0.87 ± 0.07	1.28 ± 0.12	−0.35 ± 0.13	0.010
Pyrimidine metabolism				
pseudouridine	0.95 ± 0.03	1.36 ± 0.08	−0.32 ± 0.06	<0.001
Tryptophan metabolism				
indoleacetylglycine	1.05 ± 0.11	2.62 ± 0.77	−0.64 ± 0.18	0.001
Tyrosine metabolism				
dopamine 3-O-sulfate	0.93 ± 0.06	1.67 ± 0.14	−0.54 ± 0.10	<0.001
Urea cycle; arginine and proline metabolism				
dimethylarginine (ADMA + SDMA)	0.90 ± 0.03	1.09 ± 0.03	−0.19 ± 0.04	<0.001
urea	0.87 ± 0.06	1.67 ± 0.19	−0.54 ± 0.12	<0.001
citrulline	0.87 ± 0.04	1.05 ± 0.05	−0.19 ± 0.07	0.008
**Fecal metabolites**				
Fatty acid, monohydroxy				
3-hydroxyoctanoate	1.06 ± 0.05	0.88 ± 0.05	0.21 ± 0.07	0.005
Food component, plant				
genistein sulfate	0.55 ± 0.13	0.22 ± 0.00	0.47 ± 0.16	0.005
Glutathione metabolism				
cysteinylglycine	0.94 ± 0.07	1.28 ± 0.14	−0.26 ± 0.12	0.038
Lysine metabolism				
fructosyllysine	0.93 ± 0.10	1.24 ± 0.12	−0.31 ± 0.14	0.032
Methionine, cysteine, SAM, taurine metabolism				
cystathionine	0.59 ± 0.15	1.28 ± 0.29	−0.69 ± 0.29	0.021
Phenylalanine metabolism				
phenylacetate	0.94 ± 0.12	1.77 ± 0.27	−0.54 ± 0.22	0.018

Data were analyzed via a linear mixed model with animal ID as a random variable, were rescaled to a median value of 1, and are presented as group means and standard errors; ADMA, asymmetric dimethylarginine; CKD, chronic kidney disease; SAM, S-adenosylmethionine; SDMA, symmetric dimethylarginine; SE, standard error.

**Table 4:** Differences from baseline in log-transformed data of plasma metabolites in dogs with early renal disease who consumed the low soluble fiber plus betaine and high soluble fiber plus betaine foods.

	Baseline-End of Treatment ± SE	*p*-Value
**Low soluble fiber plus betaine food**		
Leucine, isoleucine, valine metabolism		
isovalerylcarnitine (C5)	0.32 ± 0.07	<0.001
2-methylbutyrylcarnitine (C5)	0.30 ± 0.10	0.003
Medium-chain fatty acid		
palmitoleate (16:1n7)	−0.21 ± 0.10	0.043
**High soluble fiber plus betaine food**		
Acetylated peptides		
phenylacetylglycine	0.41 ± 0.19	0.037
Fatty acid, branched-chain amino acid metabolism		
propionylglycine (C3)	0.52 ± 0.18	0.005
Food component/plant		
dihydrocaffeate sulfate	1.12 ± 0.45	0.017
2-oxindole-3-acetate	0.88 ± 0.35	0.015
Leucine, isoleucine, valine metabolism		
isovalerylcarnitine (C5)	0.40 ± 0.09	<0.001
2-methylbutyrylcarnitine (C5)	0.37 ± 0.14	0.015
Long-chain polyunsaturated fatty acid (n3 and n6)		
docosatrienoate (22:3n6)	−0.58 ± 0.24	0.019
Medium-chain fatty acid		
palmitoleate (16:1n7)	−0.37 ± 0.14	0.011
5-dodecenoate (12:1n7)	−0.37 ± 0.18	0.042
Phenylalanine metabolism		
4-hydroxyphenylacetate	0.70 ± 0.25	0.007
Tryptophan metabolism		
indoleacetylglycine	0.52 ± 0.22	0.022
Tyrosine metabolism		
dopamine 3-O-sulfate	0.25 ± 0.12	0.035

SE, standard error.

**Table 5:** Correlations of plasma metabolites with serum creatinine and serum SDMA.

	Correlation with Serum Creatinine		Correlation with Serum SDMA	
	r	*p*-Value	r	*p*-Value
creatinine	0.85	<0.001	0.66	<0.001
N6,N6,N6-trimethyllysine	0.72	<0.001	0.78	<0.001
N6-carboxymethyllysine	0.71	<0.001	0.71	<0.001
pseudouridine	0.71	<0.001	0.86	<0.001
urea	0.67	<0.001	0.80	<0.001
dopamine 3-O-sulfate	0.62	<0.001	0.65	<0.001
N-acetylphenylalanine	0.62	<0.001	0.75	<0.001
2-methylbutyrylcarnitine (C5)	0.57	<0.001	0.69	<0.001
cystathionine	0.57	<0.001	0.52	<0.001
isovalerylcarnitine (C5)	0.56	<0.001	0.66	<0.001
isobutyrylcarnitine (C4)	0.55	<0.001	0.66	<0.001
hydroxyproline	0.54	<0.001	0.45	<0.001
dimethylarginine (ADMA + SDMA)	0.51	<0.001	0.71	<0.001
pyrraline	0.50	<0.001	0.58	<0.001
4-hydroxyhippurate	0.44	<0.001	0.35	<0.001
5-hydroxyindoleacetate	0.41	<0.001	0.54	<0.001
dihydroferulic acid sulfate	0.39	<0.001	0.37	<0.001
fructosyllysine	0.39	<0.001	0.51	<0.001
propionylglycine (C3)	0.39	<0.001	0.40	<0.001
indoleacetylglycine	0.36	<0.001	0.54	<0.001
N6,N6-dimethyllysine	0.30	<0.001	0.63	<0.001
3-indoxyl sulfate	0.26	0.001	0.37	<0.001
citrulline	0.26	0.001	0.42	<0.001
trimethylamine N-oxide	0.26	0.001	0.49	<0.001
docosatrienoate (22:3n6)	−0.20	0.009	−0.32	<0.001
palmitoleate (16:1n7)	−0.20	0.012	−0.35	<0.001
tetradecadienoate (14:2)	−0.26	0.001	−0.39	<0.001
docosahexaenoate (DHA; 22:6n3)	−0.30	<0.001	−0.37	<0.001
eicosapentaenoate (EPA; 20:5n3)	−0.30	<0.001	−0.46	<0.001
docosapentaenoate (DPA; 22:5n3)	−0.37	<0.001	−0.39	<0.001

ADMA, asymmetric dimethylarginine; SDMA, symmetric dimethylarginine.

**Table 6:** Proximate analyses of stools from dogs fed the three study foods.

	Study Foods			
	Control	Low Soluble Fiber plus Betaine	High Soluble Fiber plus Betaine	*p*-Value
pH	5.60 ± 0.03	5.71 ± 0.04	5.65 ± 0.04	0.095
Moisture, %	74.96 ± 0.33	74.62 ± 0.43	74.92 ± 0.41	0.810
Ash, %	4.46 ± 0.13	4.77 ± 0.14	4.64 ± 0.29	0.520

**Table 7:** Differences from baseline in log-transformed data of fecal metabolites in dogs with early renal disease who consumed the low soluble fiber plus betaine and high soluble fiber plus betaine foods.

	Baseline-End of Treatment ± SE	*p*-Value
**Low soluble fiber plus betaine food**		
Food component, plant		
genistein sulfate	−0.43 ± 0.16	0.015
Lysine metabolism		
fructosyllysine	0.30 ± 0.15	0.045
Methionine, cysteine, SAM, taurine metabolism		
cystathionine	0.65 ± 0.29	0.029
Phenylalanine metabolism		
phenylacetate	0.56 ± 0.28	0.050
**High soluble fiber plus betaine food**		
Fatty acid, monohydroxy		
3-hydroxyoctanoate	−0.20 ± 0.08	0.018
Food component, plant		
genistein sulfate	−1.53 ± 0.31	<0.001

SE, standard error.

**Table 8:** Correlations among plasma and fecal metabolites and operational taxonomic units in the fecal microbiome of dogs with early renal disease fed the control, low soluble fiber plus betaine, and high soluble fiber plus betaine foods.

Metabolite	OTU	Estimate ± SE	*p*-Value	Pearson’s Correlation Coefficient
**Plasma**				
catechol sulfate	586453 Christensenellaceae *	−0.38 ± 0.19	0.049	0.23
indolelactate	1030652 *Odoribacter*	−0.73 ± 0.32	0.026	0.26
3-methoxycatechol sulfate	586453 Christensenellaceae *	−0.40 ± 0.17	0.022	0.26
4-vinylguaiacol sulfate	1030652 *Odoribacter*	−0.49 ± 0.20	0.016	0.31
**Fecal**				
4-hydroxybenzoate	249375 Actinobacteria *	−0.35 ± 0.17	0.042	0.18
indolepropionate	249375 Actinobacteria *	−0.33 ± 0.14	0.016	0.21
Indolin-2-one	586453 Christensenellaceae *	−0.20 ± 0.08	0.010	0.23
secoisolariciresinol	249375 Actinobacteria *	0.23 ± 0.09	0.013	0.22
secoisolariciresinol	23706 *Collinsella*	0.38 ± 0.19	0.051	0.10
secoisolariciresinol diglucoside	249375 Actinobacteria *	0.25 ± 0.10	0.013	0.22

* Unclassified genus. OTU, operational taxonomic unit; SE, standard error.

## Supplementary Materials

The following are available online at https://www.mdpi.com/2218-1989/10/9/370/s1, Table S1: Eigenvectors and their corresponding eigenvalues on principal component 1 for the plasma metabolomic analysis.
